# Salivary Secretory Immunoglobulin (SIgA) and Lysozyme in Malignant Tumor Patients

**DOI:** 10.1155/2016/8701423

**Published:** 2016-05-17

**Authors:** Haiyan Sun, Yong Chen, Xuan Zou, Qihong Li, Huan Li, Yao Shu, Xia Li, Weihong Li, Li Han, Cheng Ge

**Affiliations:** ^1^Department of Stomatology, Affiliated Hospital, Academy of Military Medical Sciences, Beijing 100071, China; ^2^Center for Hospital Infection Control, Chinese PLA Institute for Disease Control & Prevention, Academy of Military Medical Sciences, Beijing 100071, China; ^3^Second Affiliated Hospital of Zhejiang University, Hangzhou, Zhejiang 310052, China

## Abstract

*Background.* The purpose of this study is to understand the oral mucosal immune status of cancer patients and to make clear whether antibacterial proteins such as salivary secretory immunoglobulin (SIgA) and lysozyme in saliva were influenced by patients' health status and certain medical treatment therapy.* Materials and Methods.* This study included 221 patients with malignant tumor receiving antineoplastic treatment and 171 age- and gender-matched healthy controls.* Results.* The results showed that patients suffering malignant tumor had lower level of SIgA and higher level of lysozyme than healthy subjects (*P* < 0.05). The SIgA level was significantly different among different cancer tumors, while the lysozyme level showed significant difference only between patients with digestive tract malignant tumor and hematopoietic system tumor. Pretreatment before transplantation for hematopoietic system tumor patients significantly affected the lysozyme level other than SIgA. SIgA level was affected by many factors such as age, therapy factors, and oral hygiene.* Conclusion.* Malignant tumor and the antineoplaston may weaken the patients' oral mucosal immunity, influence levels of some salivary proteins, and decrease the level of SIgA, resulting in aggregation of oral bacteria and failure of clearing them from the oral cavity.

## 1. Background

Saliva protects oral tissues in many ways. Normal saliva flow and phosphate buffering system can maintain the ability of self-clearance and inhibition of a large number of acid-producing cariogenic bacteria from the oral cavity. An important saliva ingredient is a group of antibacterial proteins including immunoglobulin (e.g., salivary secretory immunoglobulin A (SIgA), immunoglobulin G (IgG), and immunoglobulin M (IgM)) and nonimmunoglobulin (e.g., lysozyme, lactoferrin, lactoperoxidases, defensins, histatins, saliva peroxidase system, and lectin protein), which are closely related to local or systemic malfunction. These proteins play important roles not only in protecting the integrity of oral tissues, but also in providing clues for local and systemic diseases [1], such as breast cancer (systemic inflammation) [2] and oral cancers (local inflammation) [3]. Therefore, the use of saliva as a diagnosis has become a somewhat success story of translational research [4]. However, the saliva proteins can be affected by some physiological and pathological factors, such as psychological and hormonal status, ages, physical exercises, oral hygiene, drugs, and smoking [5].

Salivary SIgA is the primary means of measuring the “first line of defense” at the oral mucosal surface. It serves as an effector in mucosal immunity by suppression of submucosal invasion. Previous literatures have suggested an association between the levels of SIgA and risk of infection [6, 7]. As an important part of the nonspecific immune defense mechanism, lysozyme is an important component of antibacterial in saliva. It participates in the host nonimmune defense against bacteria, maintaining the steady state equilibrium of the oral cavity environment. Salivary lysozyme has been reported to be associated with hypertension [8], coronary artery disease [9], and arterial stiffness [10]. Therefore, it is of interest to detect if both the salivary SIgA and lysozyme can act as the biomarkers to monitor several cancers in clinical use. However, before application, several possible factors that may affect these proteins for biomarkers should be identified, such as the patients' age, gender, active periodontal disease, volume of unstimulated saliva, neutropenia, corticoid therapy, regular chemotherapy, use of drugs with epithelial cells toxicity, antibacterial drug use, antifungal drug use, invasive treatment, and drug mouthwash.

In this study, the subjects were cancer patients receiving antineoplastic treatment, who were in a poor immune state. We selected SIgA and lysozyme as the objects of the study aiming to understand the oral mucosal immune status of cancer patients and make clear whether these antibacterial proteins in saliva were influenced by patients' health status and certain medical treatment therapy.

## 2. Material and Methods

### 2.1. Study Population

This study was conducted from October 2012 to March 2013 in the 307th Hospital of Chinese People's Liberation Army (PLA) in Beijing, China. Two hundred and twenty-one cancer patients suffering from pulmonary cancer, digestive tract malignant tumor, and hematopoietic system tumor were consecutively recruited from the in-patient department of the Tumor Therapy Center of this hospital (approved by the Ethical Committee of the Affiliated Hospital, Academy of Military Medical Sciences).

One hundred and seventy-one age- and sex-matched healthy controls were recruited randomly from the Medical Examination Center of the hospital.

All the subjects were informed about the purpose of this study and the informed consents were obtained from them. This study was approved by the Committee for Ethics and Supervision on Human Research, Academy of Military Medical Sciences, Beijing, China.

### 2.2. Oral Examination and Data Collection

Before collecting the saliva, regular oral examination was performed by the same dentist. The cavity amount, oral hygiene statement, periodontal health condition, and symptom of oral mucosa of all these participants were checked according to the periodontitis criteria adopted by the American Academy of Periodontology [11]. The information about patients' demographics and medical treatment was collected by a trained dentist.

### 2.3. Saliva Collection

Subjects were instructed to refrain from food and water for two hours before saliva collection. All unstimulated whole saliva samples were collected between 9:00 a.m. and 11:00 a.m. by a standard procedure. In detail, subjects were seated with their head tilted forward and the saliva samples were collected for 5 min via passive drool into sterilized tubes, which were then placed on ice. Saliva samples were homogenized and clarified by centrifugation at 10000 ×g for 15 min at 4°C. Finally, the aliquots of each clarified supernatant were measured by a pipette with reading accuracy of 0.01 mL and kept at −70°C until the time for analyses.

### 2.4. Detection of Salivary SIgA and Lysozyme

Saliva samples were determined by using an enzyme-linked immunosorbent assay (ELISA) kit (Uscn Life Science, Wuhan, China). Briefly, the microtiter plate had been precoated with an antibody specific to human IgA. Standards and samples (50 *μ*L each) were then added to the wells in duplicate and incubated for 2 h at 37°C. After incubation, biotin-conjugated antibody working solution was added to each well. After washing away the unbound substances, avidin conjugated to horseradish peroxidase (HRP) working solution (100 *μ*L) was added to each well and incubated for 1 h at 37 m °C. Then a tetramethylbenzidine (TMB) substrate solution (90 *μ*L) was added to the wells for 30 min; TMB substrate color turned blue at HRP enzyme-catalyzed environment; reaction was terminated by addition of a sulphuric acid solution. The intensity of the color change is measured spectrophotometrically at a wavelength of 450 nm. Then the concentration of SIgA in the samples is determined by comparing the optical density (OD) of the samples to the standard curve.

The analysis of salivary lysozyme was performed with the same method as salivary SIgA.

### 2.5. Statistical Analysis

Mann-Whitney *U* nonparametric test or *t*-test was used for two groups of independent samples in quantitative data. ANOVA or Kruskal-Wallis test was used for multiple sets of independent samples. In order to avoid the effect of age and sex on results, the differences of SIgA and lysozyme concentration between patients and controls among different cancer groups were evaluated by covariance analyses. All differences were considered significant when *P* < 0.05. Statistical analysis was carried out using Statistica 8.0 (StatSoft, USA).

## 3. Results

### 3.1. Patients' Characteristics

Patients suffering from pulmonary cancer (*n* = 63), digestive tract malignant tumor (*n* = 86), and hematopoietic system tumor (*n* = 72), aged from 9 to 80 years, were included in this study. The demographic data were listed in Table 1.

### 3.2. The Concentration of Salivary SIgA and Lysozyme in Cancer Patients and Controls

By comparing with the control group, we found that the level of salivary SIgA was significantly lower in cancer patients than that in healthy controls (*P* < 0.001). However, the lysozyme content was higher in cancer patients than that in normal controls (*P* < 0.01) (Table 2).

### 3.3. The Concentration of Salivary SIgA and Lysozyme in Different Cancer Groups

The contents of salivary SIgA and lysozyme in patients with pulmonary cancer and digestive tract malignant and hematopoietic system tumor were detected in this study (Table 3). As a result, the content of SIgA (pulmonary cancer, 28.47 ± 2.69 *μ*g/mL; digestive tract malignant tumor, 26.87 ± 3.88 *μ*g/mL; hematopoietic system tumor, 21.41 ± 1.63 *μ*g/mL) was significantly different between any two of the three groups (*P* < 0.05). The content of lysozyme was much more in the hematopoietic system tumor group (56.13 ± 7.61 ng/mL) than that in the digestive tract malignant tumor group (52.39 ± 12.64 ng/mL) (*P* < 0.05).

### 3.4. The Concentration of Salivary SIgA and Lysozyme in Different Treatments Patients with Hematopoietic System Tumor

We then further detect if different chemotherapies for patients with hematopoietic system tumor could affect the salivary SIgA and lysozyme. The results suggested that the salivary lysozyme level was much higher in the patients receiving pretransplant chemotherapy than those receiving regular chemotherapy (Table 4). However, different chemotherapies did not significantly affect the salivary SIgA level.

### 3.5. Analysis of Possible Factors Influencing Salivary SIgA and Lysozyme in Cancer Patients

In order to identify the possible factors influencing salivary SIgA and lysozyme, we performed a covariance analysis based on age, gender, active periodontal disease, volume of unstimulated saliva, neutropenia, corticoid therapy, regular chemotherapy, use of drugs with epithelial cells toxicity, antibacterial drug use, antifungal drug use, invasive treatment, and drug mouthwash in this study (Table 5). Among these factors, only antifungal drug use was found to be related to the lysozyme level in cancer patients; it was also significantly associated with the SIgA level (*P* < 0.01). Age was found to be related to the level of salivary SIgA (*P* < 0.01). Besides, neutropenia, invasive treatment, corticoid therapy, use of drugs with epithelial cells toxicity, antibacterial drug use time, antifungal drug, and drug mouthwash all reduced the level of salivary SIgA.

It was also found that the gender, active periodontal disease, volume of unstimulated saliva, and regular chemotherapy had no influence on the levels of the two salivary proteins.

## 4. Discussion

Oral cavity plays an important barrier role of mucous membranes in addition to the physical shielding properties of epithelia and mucin. The protective function occurs via the immunoglobulins and nonimmune antibacterial proteins in saliva by aggregating the pathogenic bacteria and clearing them from the oral cavity. When some functions of the patient body are abnormal, the level of these antibacterial factors may change; then the stabilization of the oral environment will be destroyed.

In this study, we found that the levels of salivary SIgA and lysozyme in cancer patients were significantly different from those in healthy control individuals. Furthermore, the salivary SIgA was significantly different among different cancer groups, while salivary lysozyme showed significant difference only between patients suffering from digestive tract malignant tumor and hematopoietic system tumor. We then detected the concentration of SIgA and lysozyme in different chemotherapies and found that these chemotherapies significantly affected the lysozyme level other than the SIgA level. We also detected the possible factors that affect the lysozyme and SIgA level in these patients and found that SIgA was significantly associated with neutropenia, corticoid therapy, use of drugs with epithelial cells toxicity, antibacterial drug use, antifungal drug use, invasive treatment, and drug mouthwash. With regard to lysozyme, only antifungal drug use significantly associated with saliva lysozyme level.

Researchers proposed that as one of the exocrine secretions saliva could be used to monitor either oral or systemic abnormality. As the “first line of defense” at the oral mucosal surface, the salivary SIgA was found reduced in cancer patients compared to normal controls in our study. Gleeson and his partners reported an increase change in SIgA level followed with 12-week moderate exercise, and this increase was associated with reduction of sick days [12]. On the contrary, another study suggested that SIgA was significantly higher in the limited and diffused systemic sclerosis compared with the healthy control [13]. Though the changes about salivary SIgA are still controversial in low immune patients or healthy individuals, there is no doubt that salivary SIgA is associated with immune function. The present study also showed the SIgA was significantly different in different cancer types. We believe that the salivary SIgA level is a sensitive indicator for immune diseases and its diagnostic value should be explored in the future research.

With regard to lysozyme, earlier publications have reported the oral infections, hyperglycemia, hypertension, and metabolic syndrome significantly associated with increased salivary levels of lysozyme [14]. Lysozyme exerts immune activity through hydrolysis of *β*-1,4-glycosidic bonds of bacterial cell wall peptidoglycan. Besides, it also plays a key role in antiviral properties [15] and induces lysis of tumor cells [16]. Thus, the raised concentration of lysozyme in cancer patients may be explained by antitumor effect of lysozyme. In addition, the lysozyme was significantly different between patients with hematopoietic system tumor who underwent pretreatment and regular treatment. We speculate that this difference may relate to the increase of secretory immune factor by phagocytic cells because of the oral mucosal infection after chemotherapy [17]. Pretreatment before transplantation is a process to reduce the rejection reaction of receptor by suppressing the immunologic function. More prevalent oral mucous ulcer, often occurring in these patients, may stimulate local monocyte-macrophage system to secret more lysozyme [18]. All these results suggest that low immunity will result in high level of salivary lysozyme.

A covariance analysis was performed in our study in order to identify the possible factors influencing salivary SIgA and lysozyme. As a result, both SIgA and lysozyme were significantly influenced by antifungal drug use. Antitumor drug therapy often causes suppression of bone marrow, weakening of immune function, and neutropenia [19]. Some antitumor drugs can also damage the proliferation ability of oral mucosal cells and result in oral mucosal erosion and ulcer [20]. Thus these antitumor drug therapy methods may present an adverse effect on the oral environment [21]. As these patients were fragile and the infection was common, some medications such as antibiotics, antifungus drugs, and corticoid were needed for a long period. Jensen et al. [22] found chemotherapy caused a slight decrease of SIgA concentration, which remained at the same level after the end of chemotherapy in breast cancer patients. The result of this study suggested that the declining immune function because of suffering malignant tumor and related treatment may result in the decrease of salivary antibacterial ability, for instance, the decrease of salivary SIgA. We supposed that there should exist a certain relationship between changes of salivary immune proteins and cancer patients' general condition or anticancer treatment. However, the detailed relationship needs further research. Age was also found to be related to the level of salivary SIgA. It was presumed that the hematopoietic system tumor patients in this study were younger; at the same time they were immune damaged, so they showed a lower level of salivary SIgA.

There are several limitations included in this study that should be pointed out. Firstly, our study is a preliminary investigation about the relationship between antibacterial proteins in saliva and patients' health status or certain medical treatment therapy. We preliminarily concluded that malignant tumor may influence the contents of SIgA and lysozyme. However, we did not uncover the actual associations underlining them. Otherwise, the patient size is limited in this study. Only 8 cases with hematopoietic system tumor underwent pretreatment before transplantation. In addition, we did not analyze the relationship of smoking status and cancer in these patients, because cancer is highly prevalent among smokers. In order to confirm our results with more powerful evidence, large sample size and good designed trials are necessary in the further studies.

In conclusion, our preliminary investigation suggests that the malignant tumor and the antineoplaston may weaken the patients' oral mucosal immunity, influence the level of some salivary proteins such as SIgA and lysozyme, and result in decreased aggregation of oral bacteria and failure of clearing them from the oral cavity.

## Figures and Tables

**Table 1:** The characteristics of patients in this study.

	Pulmonary cancer	Digestive tract malignant tumor	Hematopoietic system tumor	*P*
Age (year)	49.48 ± 15.11	53.59 ± 11.58	38.13 ± 15.60	<0.01
Gender (male/female)	41/22	60/26	41/31	0.24

**Table 2:** The concentration of salivary SIgA and lysozyme in cancer patients receiving anticancer treatment and controls.

	Patient (*n* = 221)	Control (*n* = 171)	*P* value
SIgA (*µ*g/mL)	25.55 ± 4.18	30.27 ± 3.09	*P* < 0.001
Lysozyme (ng/mL)	53.89 ± 12.01	51.64 ± 2.82	*P* < 0.01

**Table 3:** The concentration of salivary SIgA and lysozyme in various cancer patients.

	Pulmonary cancer (*n* = 63)	Digestive tract malignant tumor (*n* = 86)	Hematopoietic system tumor (*n* = 72)
SIgA (*µ*g/mL)	28.47 ± 2.69	26.87 ± 3.88	21.41 ± 1.63
Lysozyme (ng/mL)	53.38 ± 14.74	52.39 ± 12.64	56.13 ± 7.61

For SIgA, the differences of SIgA concentration between any two patient groups were significant (*P* < 0.01). For lysozyme, there was significant difference between hematopoietic system tumor and digestive tract malignant tumor groups; the other groups showed no significant difference.

**Table 4:** The concentration of salivary SIgA and lysozyme of hematopoietic system tumor patients.

	Pretreatment before transplantation (*n* = 8)	Regular treatment (*n* = 64)	*P* value
SIgA (*µ*g/mL)	20.33 ± 3.12	21.54 ± 1.32	0.35
Lysozyme (ng/mL)	71.09 ± 16.53	54.26 ± 1.74	0.03

**Table 5:** Analysis of covariance the factors influencing salivary SIgA and lysozyme in cancer patients.

Variables	*n*	SIgA (*µ*g/mL)	*P* value	Lysozyme (ng/mL)	*P* value
Age^*∗*^					
<60	162	24.82 ± 3.96	<0.01	54.26 ± 12.28	0.45
≥60	59	27.54 ± 4.13		52.88 ± 11.27	
Gender^*∗*^					
Male	142	25.88 ± 4.23	0.13	53.89 ± 11.85	0.86
Female	79	24.95 ± 4.03		53.88 ± 12.36	
Active periodontal disease					
Yes	9	28.27 ± 6.96	0.27	59.00 ± 8.59	0.21
No	212	25.43 ± 4.00		53.67 ± 12.10	
Volume of unstimulated saliva (mL/5 min)					
<2 mL	151	25.63 ± 4.00	0.69	53.15 ± 11.70	0.19
≥2 mL	70	25.37 ± 4.63		55.49 ± 12.66	
Neutropenia					
<1.5 × 10^9^	61	22.83 ± 3.31	<0.01	53.80 ± 10.11	0.86
>1.5 × 10^9^	160	26.53 ± 3.88		53.92 ± 12.69	
Corticoid therapy					
Yes	111	24.25 ± 3.98	<0.01	54.06 ± 11.05	0.75
No	110	26.78 ± 3.77		53.72 ± 12.96	
Regular chemotherapy					
Yes	127	25.45 ± 4.07	0.82	54.00 ± 12.86	0.89
No	94	25.58 ± 4.10		53.74 ± 10.81	
Use of drugs with epithelial cells toxicity					
Yes	26	21.47 ± 2.18	<0.01	58.09 ± 12.33	0.07
No	195	26.05 ± 3.96		53.33 ± 11.89	
Antibacterial drug use ≥ 3 d					
Yes	83	23.49 ± 3.76	<0.01	54.99 ± 11.00	0.34
No	138	26.72 ± 3.77		53.28 ± 12.60	
Antifungal drug use					
Yes	55	22.25 ± 2.68	<0.01	57.57 ± 9.14	<0.01
No	166	26.58 ± 3.88		52.67 ± 12.61	
Invasive treatment					
Yes	92	23.50 ± 3.97	<0.001	55.21 ± 10.30	0.18
No	129	26.94 ± 3.52		52.95 ± 13.05	
Drug mouthwash					
Yes	47	22.67 ± 2.52	<0.001	53.85 ± 6.68	0.86
No	174	26.27 ± 4.08		53.90 ± 13.10	

^*∗*^Data was analyzed by *t*-test.
